# Optimization Study on Surface Roughness and Tribological Behavior of Recycled Cast Iron Reinforced Bronze MMCs Produced by Hot Pressing

**DOI:** 10.3390/ma14123364

**Published:** 2021-06-17

**Authors:** Aydın Güneş, Ömer Sinan Şahin, Hayrettin Düzcükoğlu, Emin Salur, Abdullah Aslan, Mustafa Kuntoğlu, Khaled Giasin, Danil Yurievich Pimenov

**Affiliations:** 1 Department of Mechanical Engineering, Abdullah Gül University, Kayseri 38080, Turkey; 2 Department of Mechanical Engineering, Konya Technical University, Konya 42075, Turkey; ossahin@ktun.edu.tr; 3 Mechanical Engineering Department, Technology Faculty, Selcuk University, Konya 42130, Turkey; hayduzcukoglu@selcuk.edu.tr (H.D.); mkuntoglu@selcuk.edu.tr (M.K.); 4 Metallurgical and Material Engineering Department, Technology Faculty, Selcuk University, Konya 42130, Turkey; esalur@selcuk.edu.tr; 5 Department of Mechanical Engineering, Faculty of Engineering, Selcuk University, Akşehir, Konya 42130, Turkey; aaslan@selcuk.edu.tr; 6 School of Mechanical and Design Engineering, University of Portsmouth, Portsmouth PO1 3DJ, UK; khaled.giasin@port.ac.uk; 7 Department of Automated Mechanical Engineering, South Ural State University, Lenin Prosp. 76, Chelyabinsk 454080, Russia; danil_u@rambler.ru

**Keywords:** composites, bronze matrix, surface roughness, tribological behavior

## Abstract

Surface roughness reflects the quality of many operational parameters, namely service life, wear characteristics, working performance and tribological behavior of the produced part. Therefore, tribological performance is critical for the components used as tandem parts, especially for the MMCs (Metal Matrix Composites) which are a unique class of materials having extensive application areas such as aerospace, aeronautics, marine engineering and the defense industry. Current work covers the optimization study of production parameters for surface roughness and tribological indicators of newly produced cast iron reinforced bronze MMCs. In this context, two levels of temperature (400 and 450 °C), three levels of pressure (480, 640 and 820 MPa) and seven levels of reinforcement ratios (60/40, 70/30, 80/20, 90/10, 100/0 of GGG40/CuSn10, pure bronze-as received and pure cast iron-as received) are considered. According to the findings obtained by Taguchi’s signal-to-noise ratios, the reinforcement ratio has a dominant effect on surface roughness parameters (Ra and Rz), the coefficient of friction and the weight loss in different levels. In addition, 100/0 reinforced GGG40/CuSn10 gives minimum surface roughness, pure cast iron provides the best weight loss and pure bronze offers the desired coefficient of friction. The results showed the importance of material ingredients on mechanical properties by comparing a wide range of samples from starting the production phase, which provides a perspective for manufacturers to meet the market supply as per human requirements.

## 1. Introduction

Copper can be utilized in many engineering fields for key industrial components in the electric, electronic, aviation and automotive sectors, such as heat exchangers, air conditioners, bearings and underground heating systems, owing to its outstanding thermal and electrical conductivity, high melting point and corrosion resistance [1,2,3,4,5,6]. In the previous work conducted by the authors, copper was used as the main material with tin and was reinforced by graphite, which has the potential in producing new material for use in bushing fabrication due to its self-lubricating characteristic [1]. It was aimed to improve the mechanical properties of copper for obtaining a porous structure that paves the way for self-lubrication. Thus, an important challenge can be solved for the machine elements operated as dual parts such as shaft and bushing. These types of parts require good surface integrity and tribological properties for elongated service conditions with high performance. The MMCs must obtain better tribological properties which will be the main topic of this paper [7,8].

To overcome the limitations of the application area of copper, mechanical properties need to be improved for better tensile strength and tribological aspects [9,10,11,12]. For this purpose, it is quite important to select the accurate reinforcements which should maintain the existential properties and improve the copper matrix [13]. A handful number of papers have been presented in the past for better-constructed composites which use copper in the first place. Graphene reinforced copper composites have been preferred by researchers [2,14,15]. Accordingly, it was determined that graphene is an excellent reinforcement material with brings along superior features. In the paper from Mai et al. [16], graphene reinforced by nickel was produced for a self-lubricating material. The findings of this study exhibited that the addition of a certain level of graphene reinforced by nickel increased the tribological properties of copper. Xiao et al. [17] introduced the effect of MoS_2_ into the copper to create pins, and measured its tribology against AISI 52100. An optimization study was performed for the determination of the amount of ideal content. Wu et al. [18] tried to add Ti_2_SnC particles characterized by improved electrical and mechanical properties. Significant improvement was observed in wear rate and friction coefficient. Ma and Lu measured the effect of sliding distance on tribological aspects of copper–graphite composite materials where friction conditions and wear mechanisms were evaluated [19]. The graphite particle size effect was evaluated for copper-based composites in a paper [20] that showed the superiority of nano-sized particles in wear resistance and friction conditions. In another work [21], graphite was added into the copper material structure to investigate the surface properties, tribology and wear mechanism. Graphene and tungsten-containing in copper matrix composites were tested for a range of loads for wear and tribological events in a research from Cao et al. [22]. Copper-based composites designed for spacecraft rendezvous were investigated to understand tribological reactions from a group of authors [23]. Tang et al. used carbon fibers for the strengthened structure of copper composites which showed better properties compared to pure copper [24]. Kumar et al. evaluated the microstructure and tribology of copper–tin and MoS_2_ composites [25]. Zhao et al. measured the coefficient of friction, wear rate and microstructures of tungsten reinforced by copper matrix composites [26]. Then, Huang et al. examined copper composites reinforced by carbon nanotubes, observing tribology and mechanical specifications [27]. Different amounts and ranges of additive particles were presented in the open literature. However, in the perspective of composite production for several purposes and high demands from them, the studies that were conducted are not sufficient today.

Some ceramics have been added to the copper-based material in the past. In a work [28], copper and graphite were selected as the base material reinforced with SiO_2_ particles for further investigation of tribological properties. A hybrid composite was fabricated by adding TiC and graphite for better tribological performance expectation by Rajkumar et al. [29]. Zhan and Zhang [30] evaluated the effect of SiC particles and graphite particulates for the determination of wear and frictional characteristic. Al_2_O_3_ ceramics were added into the copper matrix in [31,32]. Different concentrations of Al_2_O_3_ particles were added from the authors [33] and it was observed that better microstructural and tribological properties were observed. Seemingly, good improvements have been obtained in terms of strength and wear resistance with reinforcement of ceramic particles due to the load-bearing effect [30]. Gong et al. examined copper–SiO_2_ and CrC composites considering their microstructural and tribological behavior during sliding tests [34]. In brief, copper exhibited a favorable media for particle addition and improved material properties according to the open literature.

On the other hand, bronze was previously selected as the core material from a group of researchers. In a work from Valente et al. [35] graphite and bronze were used as filler materials to evaluate frictional conditions and mass loss. Miguel et al. [36] investigated bronze composites under different test conditions by adding alumina to evaluate surface roughness, wear mechanism and friction. Hanon et al. [37] worked on the tribological assessment of bronze/PLA composites for tribological and tensile tests. Wang and Yan [38] performed a study on tribological conditions of PTFE/bronze composites during sliding tests for applying several loads and test duration. Tribological performance of the PTFE-based composites was prepared and tested for research on the friction process, wear life and wear condition from a group of researchers [39]. Gao et al. focused on the tribological behavior of the tin–bronze composites considering the coefficient of friction test and wear conditions with microstructural figures [40]. As can be seen from the open literature, several reinforcements have been employed for bronze composites, however this topic still requires further investigation owing to the versatility of composite materials.

In the presented study, CuSn10 was preferred as the main matrix reinforced by GGG40 material for the production of ultimate composite samples. The work differs from the counterparts containing the special reinforcement, GGG40, to obtain upgraded properties for bronze/copper matrix composites. Additionally, the findings were evaluated by the Taguchi S/N ratio, which has not been seen before, for obtaining optimum production parameters. In this direction, 2 levels of temperature, 3 levels of pressure, and 7 levels of reinforcement ratios were encountered in the experimental tests. Best composite samples for each property and production condition were analyzed in detail.

## 2. Materials and Methods

**Figure 1:**
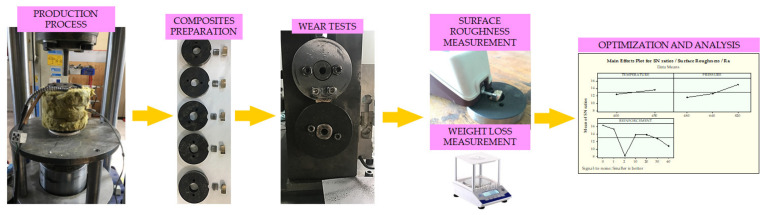
General outline of the paper.

Firstly, cylindrical CuSn10 and GGG-40 bars with 22 mm diameters were cast by Canbilenler Casting Co. Ltd., with the desired chemical composition. Later, these bars were machined by a conventional lathe technique, without using cutting fluid and they were sieved in 2-1 mm sieves, respectively, to obtain metallic chips for production. More detailed information about the hot pressing process and production stages are reported in our previous studies [41,42,43]. CuSn10 metallic chips were used as the matrix material and GGG40 as the reinforcement material in the production of MMCs used in experimental studies. These materials are widely used due to their mechanical properties. Especially CuSn10 is preferred in machine parts with its superior corrosion resistance and thermal conductivity [41]. The reinforcement material GGG40, on the other hand, is used as a lubricant material in many areas thanks to its graphite and porous structures. The chemical components of these materials are shown in Table 1 [1]. Figure 1 exhibits the general methodology of the paper including the production of composites, experimental tests, measurement of properties and their evaluation. To validate the accuracy of the data all tests were repeated three times for the same configuration. Plus, at least nine measurements were taken from surfaces for each sample to evaluate the average surface roughness.

### 2.1. Composite Materials Production Process

In the production of MMCs, 4 different mixture ratios were determined and mechanically mixed by weight in the ratios specified in Table 2. After mixing homogeneously, the hot pressing process was carried out by transferring to molds obtained from hot work tool steels. Temperature losses were minimized by providing heat isolation during the applied hot pressing process. In addition to different material components, 3 different pressures and 2 different temperatures were used during the production. With 100B, the raw CuSn10 and GGG40 materials were evaluated as reference materials and compared with the properties of the MMCs produced. After pressing, MMCs had a diameter of 19.6 mm and a length of 32–36 mm, depending on the production parameters applied. The amount of pores changed depending on the temperature and pressure applied, which changed the sample size [43].

### 2.2. Wear Tests

**Figure 2:**
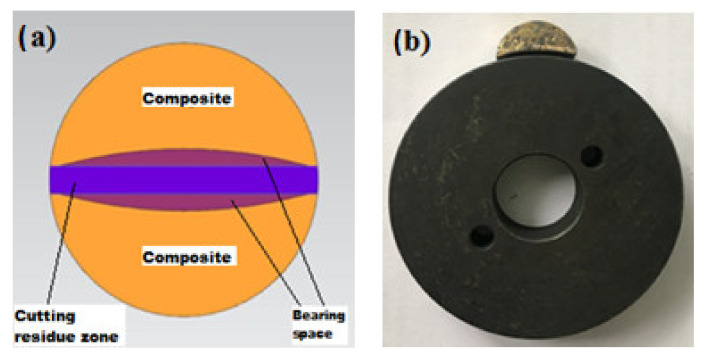
The samples used in the wear experiments: (**a**) composite materials and (**b**) abrasive disc.

After the production of MMCs, their mechanical properties were examined in detail; as a result of these investigations, it was determined that they had sufficient strength and microstructure [1,41]. Afterward, the MMCs were brought to the geometry shown in Figure 1 to perform the abrasion tests, and the appropriate bearing geometry was provided. MMCs were processed in the geometries shown in Figure 2 before wear tests to investigate their usability as a self-lubricating bearing material. These geometries have been determined for the wear of MMCs as bearing material under suitable conditions. In determining these bedding geometries, machinability features were also considered [44,45]. The cylindrical MMCs, which come out of the MMCs production molds in the specified dimensions, are sliced in a thickness of 10 mm in the diameter direction in the jigsaw, and then the sliced parts are divided in half. In the meantime, no coolant was used and care was taken to cut the MMCs properly. During cutting with the jigsaw, the purple parts shown in Figure 2a show the losses occurring due to the 2 mm thickness of the saw blade. The pink parts show the bedding geometry of the abrasive disc material. In Figure 2b, the abrasive disc material is shown by being superposed together with the MMC on which experimental studies will be conducted. Using the literature, AISI 4140 steel was selected as the abrasive disc material [46], and abrasive steel discs with a diameter of 69 mm were brought to 55–56 HRC surface hardness after cementation heat treatment. Later, surfaces of abrasive discs were made ready for experimental studies with surface grinding processes. In addition, different abrasive discs were used for each wear test. Bearing spaces with a tolerance of 0.05 mm were created inside the MMCs, which were brought to the appropriate geometries. It has been taken into consideration that the diameter of the abrasive disc material is 69 mm in forming the bearing gaps. While creating these traces, care has been taken not to damage the surface morphology of the MMCs. Finally, ultrasonic cleaning was applied to the MMCs with the desired surface structure and brought to the appropriate geometries in the CNC machining center for wear tests. This process took approximately three minutes for each sample in deionized water. Thus, it is aimed to remove unwanted residues in the wear zone [47].

**Figure 3:**
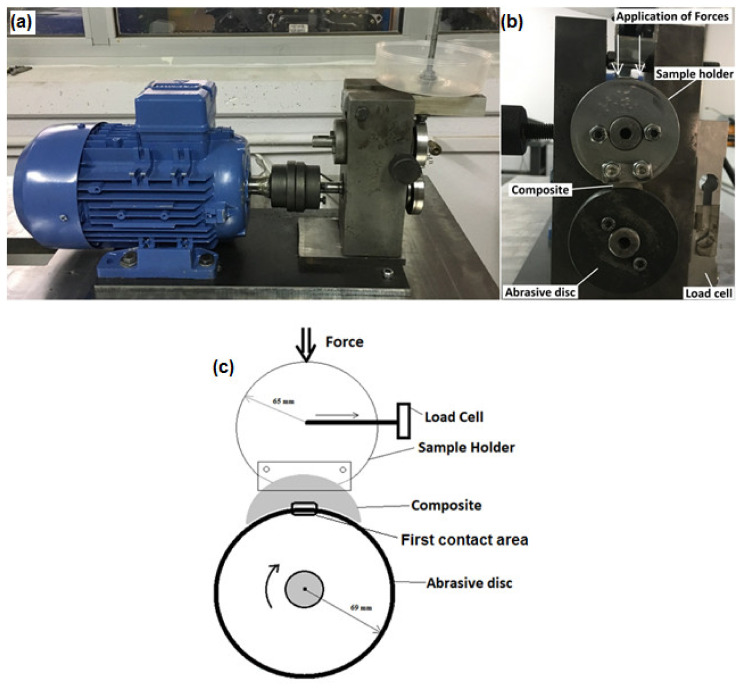
Wear test setup: (**a**) side view (**b**) front view and (**c**) schematic view.

While bringing the MMC to this geometry, a CNC was used with a thickness of 10 mm and an inner radius of 69.05 mm, and then the inner surfaces were grinded, and a suitable surface structure was obtained. Due to the macro scale of the MMC components, block-on disc wear tests were carried out on the device shown in Figure 3, observing the ASTM (G77-05) standard, to investigate the wear behavior of a certain area instead of point contact abrasion. In wear experiments, the abrasive disc speed was arranged at 400 rpm (1.06 m/s) under the 30 N load considering preliminary test results [1]; it takes approximately 31 min to complete the total wear distance of 2000 m. On the upper part of the wear test setup, there is a sample holder that provides abrasion of MMCs. The sample holder is fixed and there is a disc rotating at the bottom with a shaft powered by a 2.2 kW electric motor and with a 2.5 kW speed adjuster. The abrasive discs are made of AISI 4140 steel, which has been hardened and heat-treated as stated before, and the outer parts that will carry out the abrasion are carefully prepared. The movement of the disc occurs in the clockwise direction and the horizontal forces created by the friction force are recorded instantly on the computer through the load cell. The ratio of the vertical force applied to the horizontal friction force gives the friction coefficient. This value was regularly monitored instantly during experimental studies. In addition, the contact zone between the composite material and the abrasive disc is in the specific region as shown in Figure 3c. The friction coefficient measurements were started from the first contact area of the specimen with an abrasive disc. This area may vary depending on the environment and test conditions with the further stages of wear. In addition, no sudden changes were observed in surface roughness values thanks to the constant applied load and wear rate. The friction coefficient was measured with a Squirrel brand data logger with 8 analog inputs and recorded using the Squirrel View interface program. After three repetitions, weight losses occurring during the experimental process were determined by measuring them with precision scales before and after the experiments [1].

### 2.3. Surface Roughness Measurement

Surface roughness measurements were made with the Mitutoyo SJ-201 brand surface roughness device before and after the abrasion tests. With this device, surface roughness can be measured in materials such as hard plastic and wood as well as metals. The device consists of the main body and a portable end part. Although three different measuring distances can be adjusted, in our measurements, it is ensured that the needle tip measures the surface roughness by scanning at a distance of 2.5 mm. The needle tip and MMCs were always kept constant to avoid any inaccuracies during the measurements. Ra and Rz values were determined with the surface roughness measuring device.

### 2.4. Taguchi S/N Ratio Based Optimization

Taguchi is a design approach introduced by Genichi Taguchi years ago; it can reduce noise factors and is insensitive to variations among the process parameters [48]. The superiority of this method comes from its guaranteed high-quality design [49,50]. In general, a process contains design parameters for the experiments and response parameters for the evaluation of the results. Taguchi provides a standardized experimental design approach and uses objective functions to optimize the response parameters. The selection methodology of the objective function in Taguchi design heavily depends on the expectation from the response parameters. With respect to this, in the scope of this paper, minimum surface roughness, weight loss and coefficient of friction are desired. Therefore, in the following equation, smallest is the best titled objective function and is given as:

(1) $$S/N\;{\;}_{\mathrm{smaller}\;\mathrm{is}\;\mathrm{the}\;\mathrm{better}}=-10\log[\frac{1}{n}\;\sum_{i=1}^{n}y_{i}^{2}\;]$$

## 3. Results and Discussion

As mentioned before, 2 levels of temperature (400 and 450 °C), 3 levels of pressure (480, 640 and 820 MPa) and 7 levels of reinforcement ratios (60/40, 70/30, 80/20, 90/10, 100/0 of GGG40/CuSn10, pure bronze-as received and pure cast iron-as received) were considered in this paper for the experimental design. The full-factorial design approach was embraced for a better definition of the input parameters and determined their effect on responses. Table 3 outlines these parameters with the obtained results such as coefficient of friction-Fs, surface roughness values, arithmetical average value-Ra and average maximum-Rz and weight loss-W. A total of 42 experiments were performed and are listed in Table 3, which will be further analyzed in this section of the paper.

### 3.1. Microstructure of the Samples

**Figure 4:**
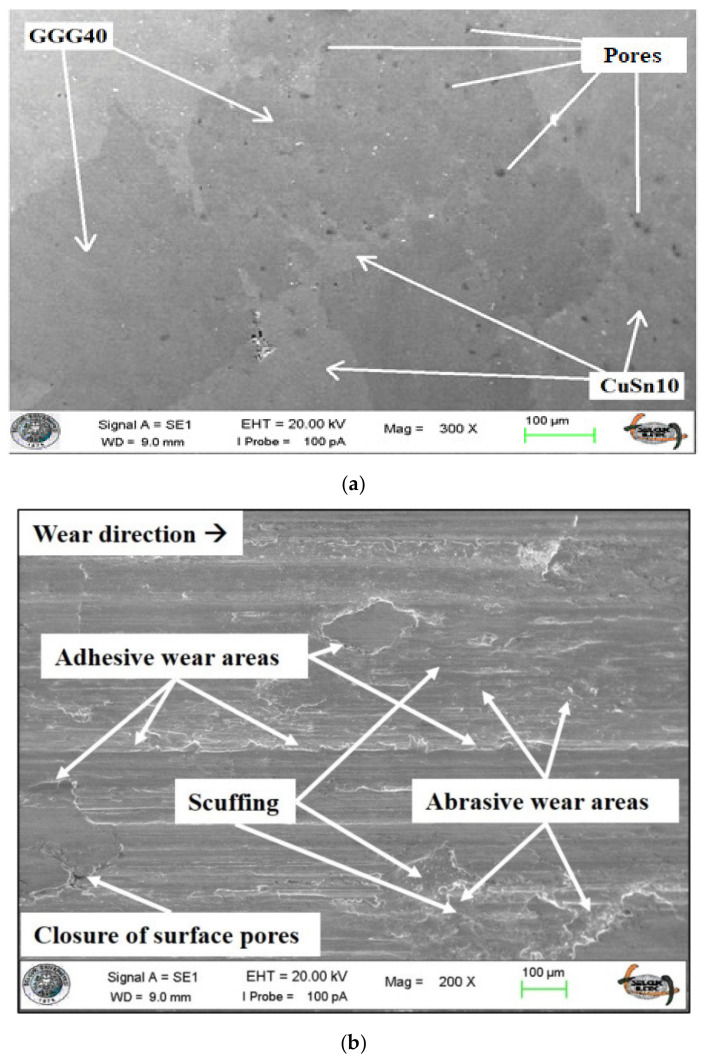

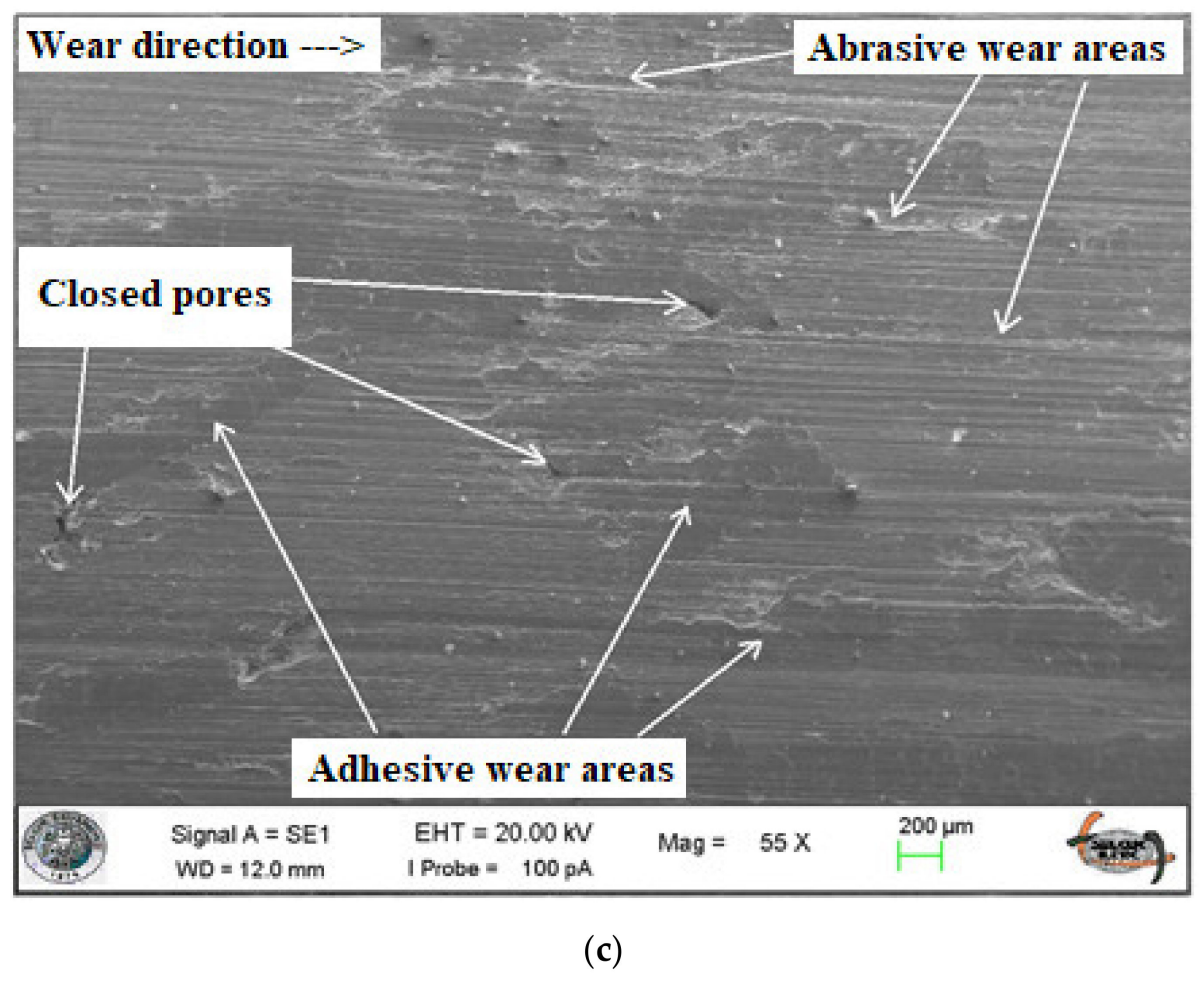
SEM image of 70B30C: (**a**) before wear, (**b**) after wear, and (**c**) SEM image of 60B40C after wear.

Figure 4 shows the SEM image of the 70B30C material produced at 820 MPa pressure before and after the wear tests. The porous structures on the surface, as well as the matrix and reinforcement material, are visible here. SEM images of the changes in the contact surface after the wear test of MMCs are shown. In all images, the wear direction is from left to right. During the wear process, heavy adhesive wear zones were observed in the contact areas. This behavior was evaluated as the coming of the abraded CuSn10 powders to the abrasion zone again due to the effect of the rotating abrasive disc and causing intense plastic deformation with the effect of increasing temperature in the contact zone [51,52].

When the wear surfaces of composite materials containing GGG40 in different proportions are examined, it is seen that the surface structure of materials containing only CuSn10 is different [41,53]. First of all, surface hardness and pore structure, which are among the most important parameters affecting the wear behavior, influenced the wear characteristics. The outcomes of our previous study [41], investigating the effect of production parameters on the Brinell hardness and porosity values, confirm these interpretations. The effects of hardness and porosity values on wear properties are explained in the following sections, and the observed results are discussed with the reported studies in the literature. In Figure 4b, there are spherical graphite structures that spread to the surface after the abrasion and the filling of the porous structures on the surface as the wear time progresses. Especially in Figure 4b, the wear areas of the composite material in the mixture ratio of 70B30C were heavily affected by the abrasive dust as shown. These areas show effective adhesive wear over time due to temperature and force applied, but there are also abrasive wear zones as indicated in the picture. These abrasive wear zones usually occur early in the composite material wear process. In this process that takes place in the running-in zone, significant wear marks are formed on the surface of the composite material. Adhesive wear zones were generally formed when the matrix material separated from the surface after running-in was plastered to the surface under the effect of temperature and pressure [43]. The surface structure of the 60B40C specimen after wear is shown in Figure 4c. It was observed that the pores of the surface were mostly covered by abrasive dust. The closure of the porous structures on the surface over time provides better heat conduction by increasing the contact area during wear. Thus, the adhesive effects are minimized by preventing the composite materials from overheating in the contact area [54,55].

**Figure 5:**
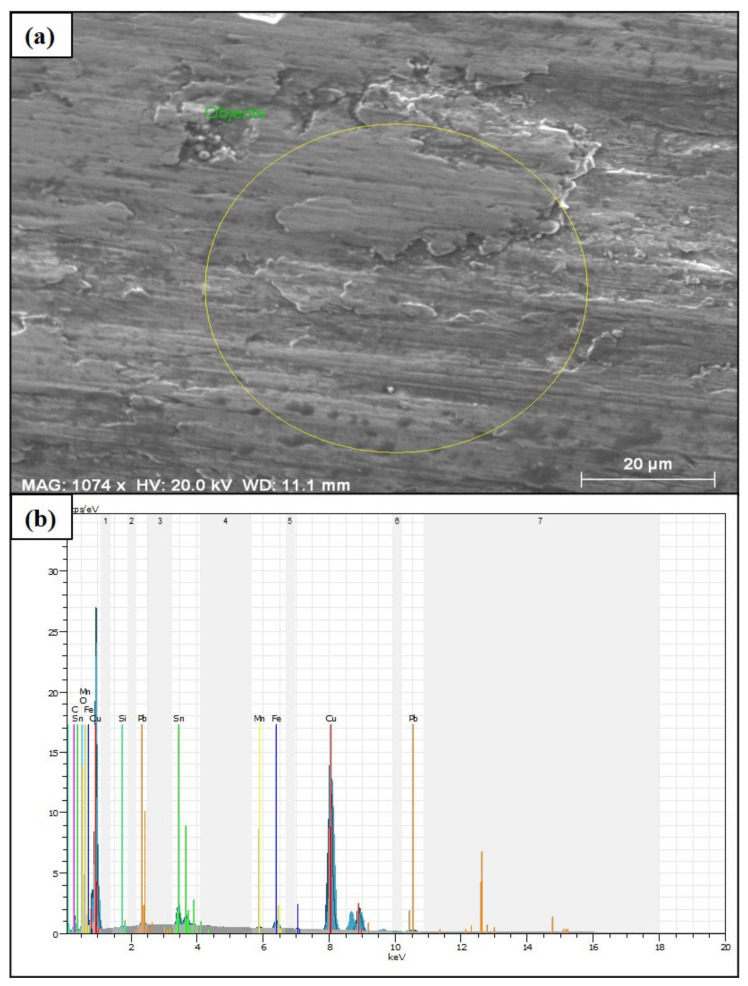
(**a**) SEM image of the 90B10C specimen after wear, and (**b**) corresponding EDX spectra of the selected region.

To monitor the variation in the chemical compositions, SEM-energy dispersive spectroscopy (EDX) analysis was performed. EDX analysis of the wear surface of the 90B10C composite produced at 820 MPa is seen in Figure 5. Figure 5a shows the area where the measurement was conducted microscopically. In the selection of this region, attention has been paid to homogeneously reflect the wear characteristics of the composite material.

Figure 5b shows the composition differences of the MMC. Within the scanned area, different alloying elements in low ratios were determined along with 83.84% copper, 5.48% tin, 1.85% iron and 1.90% graphite by weight. In addition, the detected 2.40 wt% oxidation shows that the composite material is affected by the oxygen in the environment during wear. The presence of this oxidation is expected to occur in abrasion tests conducted under room conditions. The alloying elements in the composite material must be distributed homogeneously into the matrix during production. Hence, the wear behavior of the composite material can be evaluated entirely. In the absence of this circumstance, different material behaviors are observed in the contact areas, which negatively affects the service life of the product [56]. The compatibility of copper and tin ratios on the corroded surface of the composite material with the initial conditions indicates that the surface abrasion is homogeneous. Especially, the effect of reducing the friction coefficient of graphite makes it one of the most preferred materials for bearing materials used in machine parts. In addition, it can be said that minor abrasion occurs on the surface of the abrasive disc material, which is evident by the presence of iron detected on the surface of the composite material.

### 3.2. Parameter Optimization for Surface Roughness

Optimal surface roughness values are another critical factor for part quality and energy consumption [57,58]. In other words, both “low” and “high” roughness can be detrimental for special systems. The machined surface quality is essential to determine the available workpiece operation range and performance, such as tensile strength, fatigue life, and tribological behaviors. For instance, it is well known that higher surface roughness adversely affects machined products’ fatigue performance [59]. However, the influence of surface roughness on the tribological behavior is a complicated issue, and it is not proved that lower surface roughness is good or bad for specific tribological systems [60]. For this reason, the inspection of the product quality is significant. It can be obtained by evaluating and optimizing the surface roughness parameters and other material properties. The mechanics behind the surface quality are very dynamic, complex, and process-induced variables, so it is challenging to measure their values through theoretical analyses. Hence, researchers and manufacturers frequently utilize “trial and error” approaches to obtain the preferred surface roughness. In this context, to achieve optimal tribological properties, this study adopted an experimental and statistical approach comprising of the effect of surface roughness and microstructures and their correlation between tribological aspects. To verify the accuracy of the tests and interpret the results more precisely, the surfaces of all samples were prepared in a range of 0.15–0.18 µm by grinding before the wear tests.

**Figure 6:**
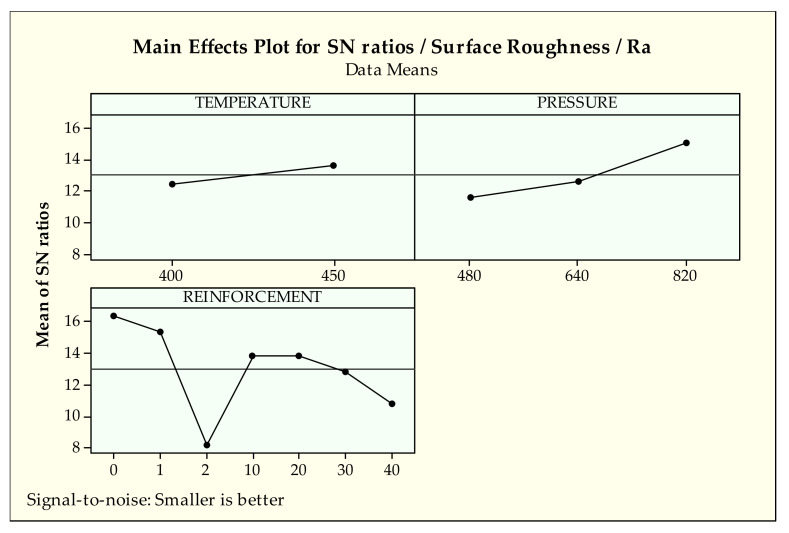
S/N ratios of surface roughness-Ra.

**Figure 7:**
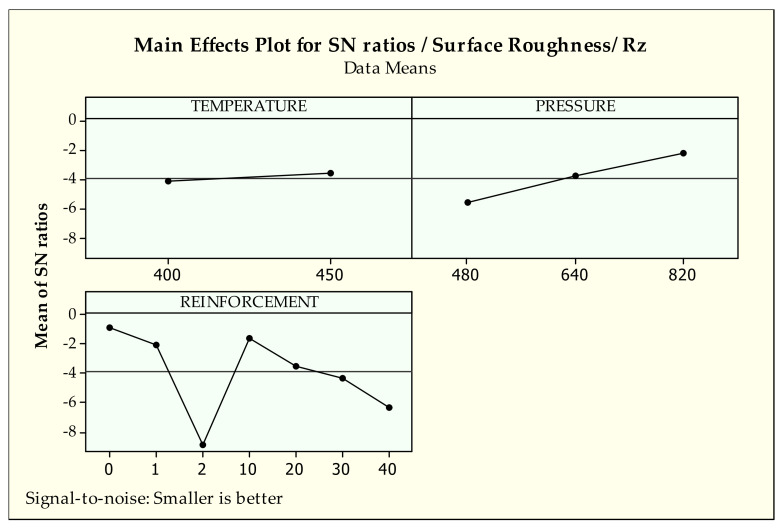
S/N ratios of surface roughness-Rz.

Figure 6 and Figure 7 demonstrate the signal-to-noise ratios of findings of Ra and Rz surface indicators, respectively. Since the two values have the same tendencies according to the design parameters, they were evaluated together. When the general trend of the parameter levels are identified, it can be said that higher S/N ratio values give the best level. Considering Figure 6 and Figure 7, it can be stated that there is a common effect of production parameters on the surface properties. In other words, as the production parameters, i.e., pressure and temperature, are increased, the surface roughness values are decreased. This observed phenomenon is attributed to better interfacial bonding quality between metallic chips due to increased production parameters [43]. The elevated pressure and temperature have positive effects on the metallic chips’ softening and plastic deformation mechanism. These circumstances supply successful penetration of the tin bronze chips into cast iron chips, resulting in good structural integrity in the MMCs systems.

When the effect of the reinforcement ratio is taken into consideration it is seen that its effects on surface roughness values (Ra, Rz) are more influential than other production parameters. Therefore, it can be concluded that the reinforcement ratio is regarded as the most influential factor on the surface quality. However, the same tendency cannot be seen for the effect of temperature and pressure. As shown in both Figure 6 and Figure 7, increasing reinforcement ratio in the MMC system causes large voids and a resultant rougher surface since cast iron chips relatively preserve their initial shape during the manufacturing [42]. When the sample came into contact with the disc during the wear test, the cast iron chips were broken and ruptured from the surface due to the formation of stress concentration at the grain boundaries between bronze and iron chips, resulting in a rougher surface [61]. These observations are well in agreement with ANOVA results. Basavarajappa et al. [62] reported the same situation for surface characteristics due to multiple phase structures of MMCs.

In addition to S/N ratios, data analysis is carried out for the determination of the effect of production parameters; analysis of variance (ANOVA) based evaluation is performed. The main advantage of ANOVA is providing many statistical parameters including percent contribution (PC %), F-value and *p*-value. Each of them brings important information about the significance of the design parameters. The PC value can be calculated by dividing each sum of square value of parameters by the total sum of square value. The F-value also demonstrates the effectiveness of factors on responses; it is calculated by dividing the sum of squares by the total value. In addition, the *p*-value evaluates the importance of the inputs in the confidence interval (95%), which should be under 5%.

According to the analysis of Ra results from Table 4, control factors, especially temperature (2.8%), have a minor effect on the surface roughness. Hence, it is very arduous to decide which control factor is more influential on surface characteristics due to contribution percentages of control factors and the high error rates of analysis. On the other hand, reinforcement (52.6%) and pressure (16.5%) have a valuable impact on the arithmetical average value of surface roughness according to percent contribution and *p*-values (0.000, 0.001 < 0.05). For the average maximum value Rz, similar effects for each parameter are observed. According to Table 5, reinforcement has a major effect (68.5%) followed by pressure (18.1%) and temperature (0.9%) respectively on Rz. Similar influences can be observed by *p*-values (0.000 for both reinforcement and pressure) on Rz.

### 3.3. Parameter Optimization for Weight Loss

**Figure 8:**
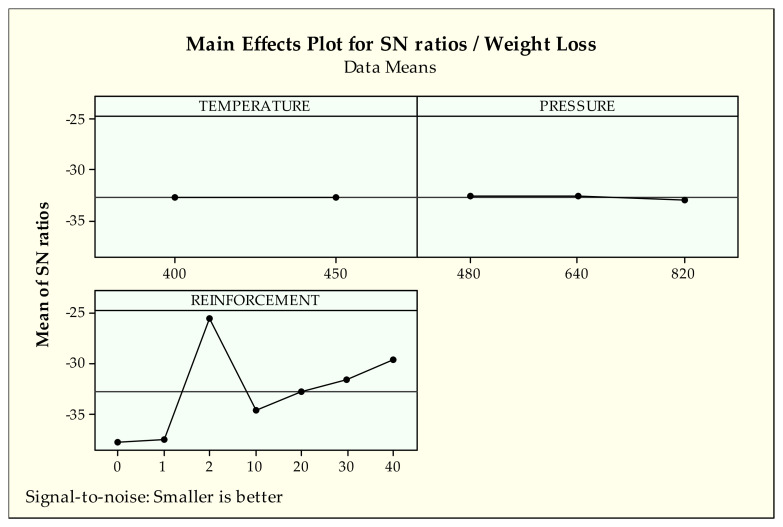
S/N ratios of weight loss.

It can be observed that in Figure 8 the first levels of temperature and pressure (400 °C and 480 MPa, respectively) produce lower weight loss. When examining the effect of reinforcement of pure cast iron (2) gives the best value while pure bronze [41] (1) and no reinforcement (0%) provide the worst results for weight loss. Seemingly, from a 10 to 40 reinforcement ratio, better results are obtained.

According to Figure 8, the pure cast iron sample exhibits the lowest weight loss compared to all samples after the wear test. The main reason for this action is that the initial iron chips are significantly harder than bronze chips. The experimental results of our previous study [1] examining the mechanical properties also support this assumption. As the proportion of the reinforcement ratio in the structure increased, the number of metallic chips removed from the surface decreased, resulting in lower weight loss. However, other production parameters, namely temperature, and pressure cannot commit such effect in this MMCs system.

Since the interfacial bonding quality [63] and variation of the amount of the pores [64,65] in the structure directly or indirectly affects the wear performance of the material system, there might be other effective parameters impacting on the weight loss which were not investigated in this study, such as hot pressing time and abrasive wear disc type.

According to Table 6, reinforcement (98.1%) has the dominant effect on weight loss compared to temperature (0%) and pressure (0.1%) in terms of PC. Temperature and pressure have a negligible effect on weight loss. Accordingly, reinforcement is the only effective parameter among others according to *p*-value (0.000 < 0.05). F-values also confirm the obtained results. These findings support the optimization plots of S/N ratios in Figure 8. Since the number of levels are higher than the others, the reinforcement effect intensifies in the selected range of the parameters.

### 3.4. Parameter Optimization for Coefficient of Friction

**Figure 9:**
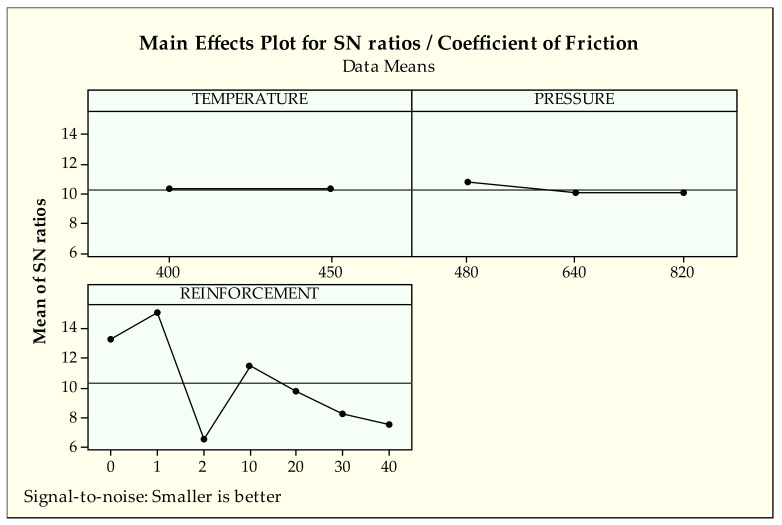
S/N ratios of coefficient of friction.

As it is seen in Figure 9, the first levels of pressure, 480 MPa, and temperature, 400 °C, seem to be the best choice for the coefficient of friction according to the main effects plot for S/N ratios. For the influences of reinforcement, it can be said that pure bronze (1) provides the most proper coefficient of friction conditions followed by the no reinforcement ratio (0%). After those effects, from 10 to 40 additions, reinforcement makes a negative influence on the coefficient of friction.

Regarding the coefficient of friction, the running-in zone is formed at 160–210 m sliding distances, which varies depending on the reinforcement ratio. Running-in is the process of removing peaks or hollows on surfaces by pre-eroding at low load and speed to determine the actual wear behavior of materials. The adhesive wear zones shown in Figure 4 are generally formed as a result of smearing of the worn part of the material on the surface with the effect of temperature and pressure after running-in [66]. Similar to deductions from weight loss results, the effect of reinforcement ratio is significantly greater than other production parameters. With the increment of reinforcement ratio, the obtained COF values increase as expected due to harder reinforcement material than the matrix. Also, it has been reported that surface pore structure and hardness play an essential role in COF values [67]. Considering the density and hardness values in our previous study [41], it is a typical situation that the COF values exhibit such a trend. In addition, according to the effect of the reinforcement ratio in both Figure 6 and Figure 9, it can be interpreted that there is a strong relationship between surface roughness and COF values. Such an interaction has been reported in different studies in the literature [68,69].

According to analysis results in Table 7, PC evaluation shows that reinforcement (92.8%) is the most dominant factor while temperature (0%) and pressure (1.1%) provide no significant contribution to the coefficient of friction. The F-value also confirms the findings of PC. In addition, *p*-values indicate that reinforcement is an important factor in the coefficient of friction (0.000 < 0.05). Considering the low error value of percent contribution, it can be concluded that reinforcement has a high-level effect on the coefficient of friction with high reliability. Also, these findings validate that the results belong to S/N ratios of optimization.

## 4. Conclusions

Tribological performance of a produced part gives important signs about the in-service conditions. Many engineering parts employed works under a tribological medium where there is exposure to the material’s harsh contact conditions. Therefore, it is critical to measure the indicators of tribological behavior of the components. In this paper, bronze metal matrix composites produced by hot pressing are evaluated in terms of the optimization point of view using the Taguchi method. In this perspective, surface roughness parameters, weight loss and coefficient of friction were handled as response parameters while reinforcement ratio, pressure and temperature were taken into account as production parameters. The concluding remarks are:

1. The reinforcement ratio dominantly affects the surface roughness parameters, Ra and Rz, weight loss and the coefficient of friction according to both S/N ratios and statistical analysis. This is an expected result because the tribological performance heavily depends on the material structure.
2. The pressure seems significant in terms of surface roughness owing to its effect on the interfacial bonding between particles during the production process. In addition, higher levels of pressure and temperature produce better surface roughness because of the same reason.
3. The obtained findings reveal that a 10% reinforcement ratio gives better surface quality and coefficient of friction, however worse weight loss, compared to higher reinforcements. No reinforcement ratio provides the lowest surface roughness; pure cast iron provides better weight loss and pure bronze provides the best coefficient of friction value.
4. Especially in the production of MMCs, where there are many variable parameters, the selection and application of the appropriate production parameters are one of the issues that should be considered, as those parameters affect the end product’s properties.

## Figures and Tables

**Table 1:** Chemical composition of the matrix and reinforcement materials at wt% [1].

Materials	C	Si	Mn	S	Mg	P	Cu	Sn	Zn	Pb
**CuSn10**	-	-	-	-	-	-	89.2	9.3	0.41	0.01
**GGG40**	3.4	2.5	0.13	0.01	0.046	0.08	-	-	-	-

**Table 2:** Composite material production parameters.

Specimen Code	Mixture Weight Ratio (wt%)	Temperature (°C)	Pressure (MPa)
60B40C	%60 CuSn10-%40 GGG40	400, 450	480, 640, 820
70B30C	%70 CuSn10-%30 GGG40	400, 450	480, 640, 820
80B20C	%80 CuSn10-%20 GGG40	400, 450	480, 640, 820
90B10C	%90 CuSn10-%10 GGG40	400, 450	480, 640, 820
100B	%100 CuSn10-%0 GGG40	400, 450	480, 640, 820

**Table 3:** Experimental results.

Experiment Number	Specimen Code	Temperature T (°C)	Pressure P (MPa)	Reinf. R (wt.%.)	Coef. of Fric. Fs (μ)	Surface R. Ra (µm)	Surface R. Rz (µm)	Weight Loss W (mg)
1	60B40C	400	480	40	0.378	0.39	2.78	29.4
2	70B30C	400	480	30	0.364	0.33	2.37	37.7
3	80B20C	400	480	20	0.346	0.27	1.97	41.9
4	90B10C	400	480	10	0.259	0.20	1.44	53.0
5	100B	400	480	0	0.222	0.18	1.28	84.2
6	Pure CuSn10	400	480	1	0.177	0.17	1.26	74.6
7	Pure GGG40	400	480	2	0.469	0.39	2.77	18.8
8	60B40C	400	640	40	0.413	0.36	2.59	34.4
9	70B30C	400	640	30	0.377	0.28	1.99	36.3
10	80B20C	400	640	20	0.300	0.22	1.56	40.6
11	90B10C	400	640	10	0.253	0.60	1.17	50.5
12	100B	400	640	0	0.246	0.15	1.10	76.6
13	Pure CuSn10	400	640	1	0.177	0.17	1.26	74.6
14	Pure GGG40	400	640	2	0.469	0.39	2.77	18.8
15	60B40C	400	820	40	0.442	0.25	1.80	30.2
16	70B30C	400	820	30	0.368	0.17	1.24	39.3
17	80B20C	400	820	20	0.313	0.15	1.13	43.1
18	90B10C	400	820	10	0.323	0.14	1.02	55.6
19	100B	400	820	0	0.216	0.13	0.94	74.7
20	Pure CuSn10	400	820	1	0.177	0.17	1.26	74.6
21	Pure GGG40	400	820	2	0.469	0.39	2.77	18.8
22	60B40C	450	480	40	0.422	0.35	2.53	26.1
23	70B30C	450	480	30	0.357	0.27	1.97	33.3
24	80B20C	450	480	20	0.305	0.29	2.07	42.9
25	90B10C	450	480	10	0.193	0.25	1.80	56.6
26	100B	450	480	0	0.184	0.21	1.50	82.8
27	Pure CuSn10	450	480	1	0.177	0.17	1.26	74.5
28	Pure GGG40	450	480	2	0.469	0.39	2.77	18.8
29	60B40C	450	640	40	0.405	0.23	1.63	27.4
30	70B30C	450	640	30	0.418	0.19	1.37	35.3
31	80B20C	450	640	20	0.351	0.18	1.34	43.6
32	90B10C	450	640	10	0.282	0.17	1.22	53.6
33	100B	450	640	0	0.260	0.14	1.05	76.2
34	Pure CuSn10	450	640	1	0.177	0.17	1.26	74.5
35	Pure GGG40	450	640	2	0.469	0.39	2.77	18.8
36	60B40C	450	820	40	0.470	0.20	1.44	35.6
37	70B30C	450	820	30	0.434	0.17	1.21	45.2
38	80B20C	450	820	20	0.344	0.15	1.12	47.7
39	90B10C	450	820	10	0.312	0.10	0.76	53.6
40	100B	450	820	0	0.185	0.12	0.86	71.5
41	Pure CuSn10	450	820	1	0.177	0.17	1.26	74.6
42	Pure GGG40	450	820	2	0.469	0.39	2.77	18.8

**Table 4:** Analysis of variance for S/N ratios of surface roughness (Ra).

Source	Degree of Freedom	Sum of Squares	Mean Square	F-Value	*p*-Value	Percent Contribution (%)
Temperature	1	14.76	14.759	3.19	0.083	2.8
Pressure	2	87.69	43.845	9.49	0.001 ^a^	16.5
Reinforcement	6	278.18	46.364	10.03	0.000 ^a^	52.6
Residual error	32	147.88	4.621	-	-	27.9
Total	41	528.51	-	-	-	-

^a^ 95% confidence interval.

**Table 5:** Analysis of variance for S/N ratios of surface roughness (Rz).

Source	Degree of Freedom	Sum of Squares	Mean Square	F-Value	*p*-Value	Percent Contribution (%)
Temperature	1	3.938	3.938	2.36	0.134	0.9
Pressure	2	77.925	38.962	23.36	0.000 ^a^	18.1
Reinforcement	6	294.587	294.587	49.098	0.000 ^a^	68.5
Residual error	32	53.375	1.668	-	-	12.4
Total	41	429.825	-	-	-	-

^a^ 95% confidence interval.

**Table 6:** Analysis of variance for S/N ratios of weight loss.

Source	Degree of Freedom	Sum of Squares	Mean Square	F-Value	*p*-Value	Percent Contribution (%)
Temperature	1	0.001	0.001	0.00	0.961	0
Pressure	2	1.229	0.615	1.66	0.206	0.1
Reinforcement	6	689.959	114.993	310.53	0.000 ^a^	98.1
Residual error	32	11.850	0.370	-	-	1
Total	41	703.039	-	-	-	-

^a^ 95% confidence interval.

**Table 7:** Analysis of variance for S/N ratios of coefficient of friction.

Source	Degree of Freedom	Sum of Squares	Mean Square	F-Value	*p*-Value	Percent Contribution (%)
Temperature	1	0.001	0.0006	0.00	0.977	0
Pressure	2	4.438	2.2190	3.13	0.057	1.1
Reinforcement	6	351.917	58.6529	82.72	0.000 ^a^	92.8
Residual error	32	22.690	0.7091	-	-	5.9
Total	41	379.046	-	-	-	-

^a^ 95% confidence interval.

## Data Availability

Not applicable.
